# Inter-screw index as a novel diagnostic indicator of tether breakage

**DOI:** 10.1007/s43390-023-00679-w

**Published:** 2023-04-04

**Authors:** Sandra H. T. Wan, Ogulcan Guldeniz, Matthew H. Y. Yeung, Jason P. Y. Cheung, Kenny Y. H. Kwan, Kenneth M. C. Cheung

**Affiliations:** 1 Department of Orthopaedic Surgery, HKU-Shenzhen Hospital, Shenzhen, China; 2 Department of Orthopaedics and Traumatology, The University of Hong Kong, Pokfulam, Hong Kong SAR, China

**Keywords:** Vertebral body tethering, Adolescent idiopathic scoliosis, Tether breakage

## Abstract

**Purpose:**

Tether breakage is the most common complication of Vertebral Body Tethering (VBT) occurring in up to 52% of Adolescent Idiopathic Scoliosis (AIS) patients and risks continued progression and revision. Radiographical diagnosis of tether breakage is commonly defined by a 5° increase in inter-screw angle and associates breakage with loss of correction. However, the sensitivity of this method was 56% only, suggesting that tethers can break without an increase in angulation, which was supported by other studies. To our knowledge, current literature lacks a method merely focusing on the diagnosis of tether breakage radiographically that does not associate the breakages with loss of correction.

**Methods:**

This was a retrospective review of prospectively collected data of AIS patients who underwent VBT. The “inter-screw index” is defined as the percentage increase in inter-screw distance since post-op, with ≥ 13% increase defined as tether breakage as suggested by our mechanical tests. CTs were reviewed to identify the breakages and compared with inter-screw angle and inter-screw index.

**Results:**

94 segments from 13 CTs were reviewed, and 15 tether breakages were identified. Use of inter-screw index correctly identified 14 breakages (93%), whereas ≥ 5° increase in inter-screw angle only identified 12 breakages (80%).

**Conclusion:**

Use of inter-screw index is proven to be more sensitive than inter-screw angle in identifying tether breakages. Therefore, we propose the use of inter-screw index to diagnose tether breakages radiographically. Tether breakages were not necessarily accompanied by a loss of segmental correction leading to an increase in inter-screw angle, especially after skeletal maturity.

**Level of evidence:**

Level 3.

## Introduction

Vertebral Body Tethering (VBT) has gained popularity in recent years as a non-fusion growth modulation method for treating scoliosis in skeletally immature patients [1]. Despite all its benefits over the traditional methods, complications of VBT are well acknowledged throughout the literature. In their recent meta-analysis, Shin et al. reported the pooled complication rate and the revision rate as 26 and 24.7%, respectively [2]. Similarly, a multi-center study by Abdullah et al. showed a 15.8% pooled complication rate by 2-year follow-up, which included thoracoscopy-related pulmonary conditions, tether breakage, and curve progression [3].

Tether breakage was reported as the most common complication of VBT and reported to be risking continued progression and subsequent revision if it occurs prior to skeletal maturity [2]. Furthermore, a breakage would ultimately mean the segmental compression through the tether is lost, which means that curve correction may not continue even if there is no loss of correction. Moreover, the particulate debris generated at the breakage area is reported to be a potential source of complications that are yet to be identified [4, 5]. As the tether breakage is a potential complication of the VBT that may significantly influence the surgical outcome, accurate diagnosis of it becomes essential [6, 7].

Tether breakage was reported to be occurring in up to 3–52% of patients, where a huge variation between the reported breakage rate data throughout the literature was observed [7–10]. Raitio et al. reported that due to this considerable variation between different studies, the true complication rates of VBT remain to be established [11]. An increase of 5-degree coronal angulation on the inter-screw angle between any two adjacent levels was the generally accepted criterion to diagnose the tether breakage radiographically throughout the literature as visual confirmation radiographically is not possible due to the radiopaque nature of the tether [7–9, 12]. However, this method has shown to be only 56% accurate in identifying the tether breakages, which could explain the variation between the breakage rates throughout the literature [13].

Inter-screw angle criterion, which is an index derived from the Cobb angle method, associates the tether breakage with loss of correction. However, recent studies showed that the loss of correction may not always be an indicator of the tether breakages, and explain why a change in inter-screw angle is recognised to underdiagnose tether breakages. Trobish et al. reported the poor accuracy of this method in diagnosing the tether breakages, and showed that tether breakage was not always accompanied by loss of correction [7]. Similarly, Baroncini et al. showed that other factors, such as the timing of the breakage, could influence the correlation between the tether breakage and the loss of correction significantly [6]. To the authors’ knowledge, the current literature lacks a method merely focusing on the diagnosis of the tether breakage radiographically that does not associate the breakages with loss of correction.

Previously, we performed tensile tests on single unit VBT constructs (REFLECT™, Scoliosis Correction System, Globus Medical) and investigated the structural mechanics and the failure mechanisms of the VBT. Our results showed that the inter-screw distance can only increase up to 10–13% prior to failure, which corresponds to an elongation on the length of PET tether. We reported that the inter-screw distance has the potential to be the bridge between mechanical investigation and radiographical examination as the measurement of elongation between the post-op and follow-up radiographs is possible, and suggested its use to diagnose the tether breakages radiographically over inter-screw angle method. However, further clinical validation was necessary to establish its reliability [14].

The aim of this study was to derive a tether breakage diagnosis index employing the inter-screw distance, the inter-screw index, assess its sensitivity compared to inter-screw angle, and to validate it based on confirmed breakage on CT scans. To the authors’ knowledge, this is the first study providing an alternative method to diagnose tether breakages radiographically which is developed based on mechanical investigation and failure mechanisms of VBT.

## Material and methods

### Inter-screw index & inter-screw angle

**Fig. 1:**
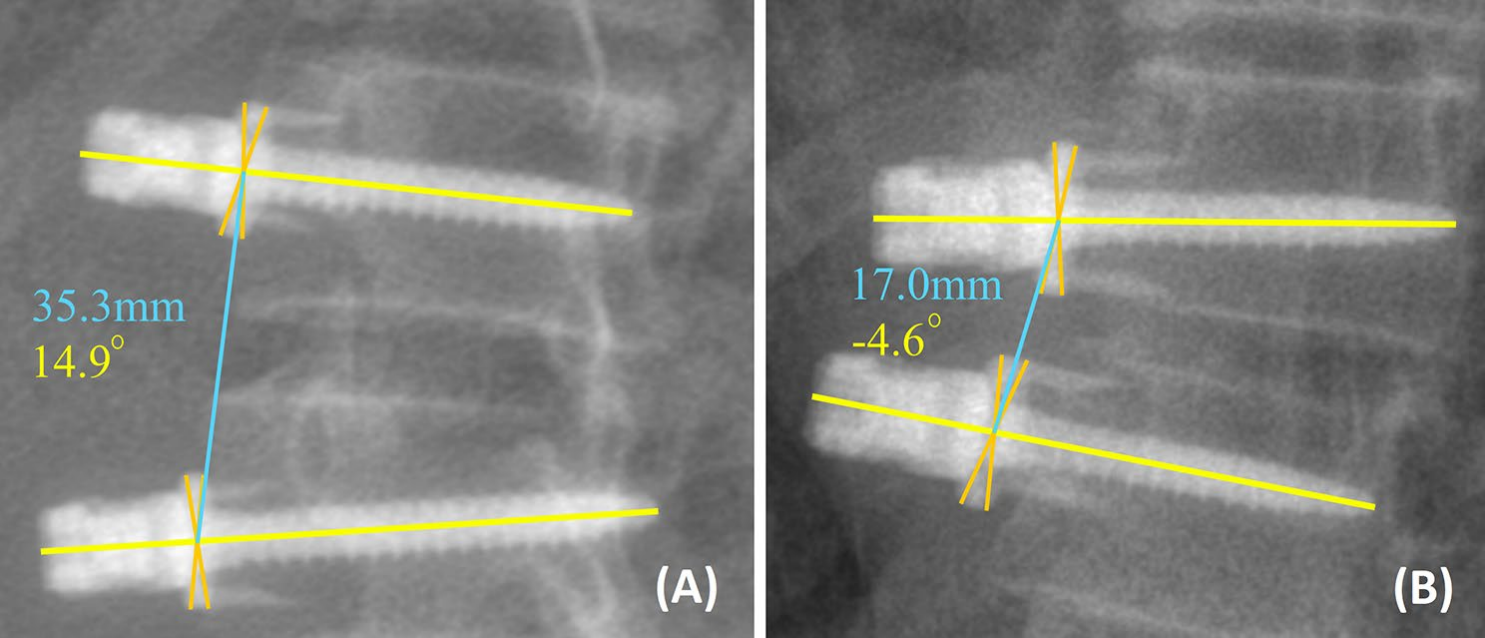
Measurement of inter-screw distance and inter-screw angle on coronal radiographs. Orange lines illustrates the diagonals of the staples, and the blue line is the inter-screw distance line connecting the intersections of the diagonals of adjacent staples (**A** and **B**). Yellow lines illustrate the screw axis, where the angle between the two adjacent screw axes represents the inter-screw angle (**A** and **B**). Shown on (**A**), concave angles are denoted as positive (+). Shown on (**B**), convex angles are denoted as negative (–)

**Fig. 2:**
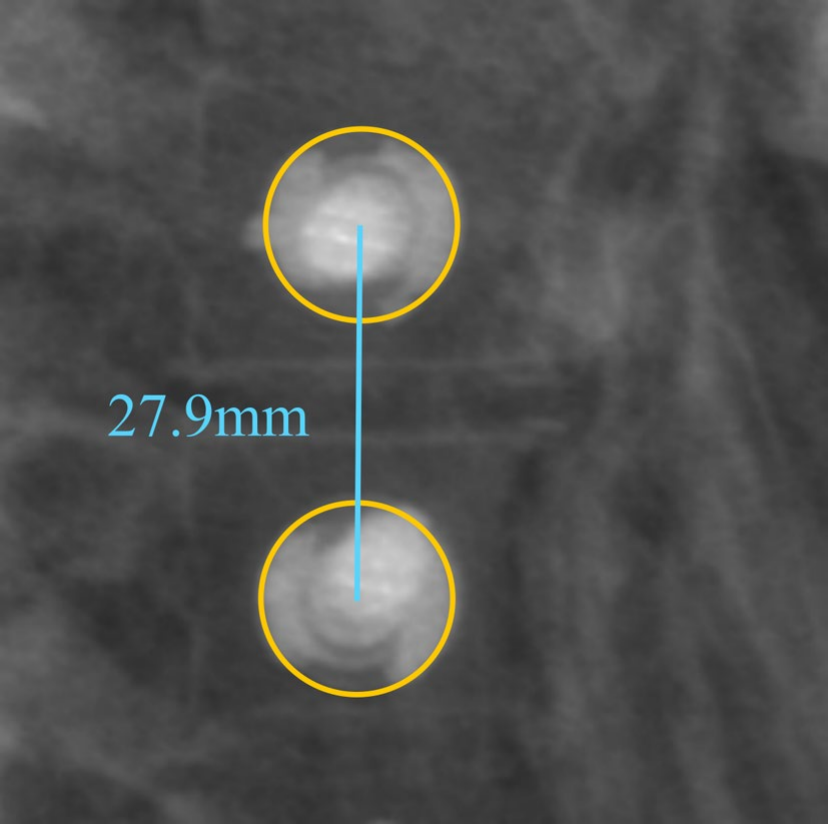
Measurement of inter-screw distance on sagittal radiographs. Centers of the orange circles surrounding the two adjacent screw heads were used to draw the inter-screw distance line

On coronal radiographs, inter-screw distance was measured as the distance between the centers of the staples of two adjacent screw heads, where centers of the staples were geometrically located as the intersection of the diagonals of the staples. On sagittal radiographs, the distance between the centers of the circles surrounding the two adjacent screw heads were used to measure the inter-screw distance (Figs. 1 and 2). Inter-screw index is defined as the percentage change in inter-screw distance between the latest follow-up radiograph and postoperative first standing radiograph. As inter-screw distance can be measured on both coronal and lateral radiographs, the larger change in distance in either plane is used for computing the inter-screw index. As observed in our mechanical studies, the maximum elongation limit of the tether prior the breakage, 13%, was used to define the inter-screw index threshold for the diagnosis of the tether breakage [14] (Eq. 1).

Equation 1: Inter-screw index.

1 $$\frac{\mathrm{Latest\, inter}\hbox{-}\mathrm{screw\, distance}-\mathrm{ Post}\hbox{-}\mathrm{op\, inter}\hbox{-}\mathrm{screw\, distance}}{\mathrm{Post}\hbox{-}\mathrm{op\, inter}\hbox{-}\mathrm{screw\, distance}} \times 100\% \ge 13\mathrm{\%}.$$

Inter-screw angle was measured as the angulation between two adjacent screw axes on coronal radiographs, with convex angles as negative (–) and concave angles as positive (+) (Fig. 1). An increase in angle of more than or equal to $5^\circ$ was defined as the conventional criteria for breakage based on its widespread use in the current literature [7, 9].

### CT imaging parameters & diagnosis of tether breakage

Toshiba Aquilion ONE and PRIME CT Scanners (Toshiba Corporation, Tokyo, Japan) were used for the collection of CT scans. The resolution of the images as well as the other CT scanning settings were modulated by the Automatic Exposure Control (AEC) system, which is a system aiming to maximize the image resolution while minimizing the radiation exposure, and is a part of the regular practice in our clinic [15]. On average, the slice thicknesses, pixel sizes, peak voltage, and the X-ray tube current of the CT scans used in this study were 1.2 ± 0.7 mm, 0.38 ± 0.1 mm, 117 ± 7 kV, and 119 ± 35 mA, respectively. No additional filters were used to attenuate metallic artefacts.

**Fig. 3:**
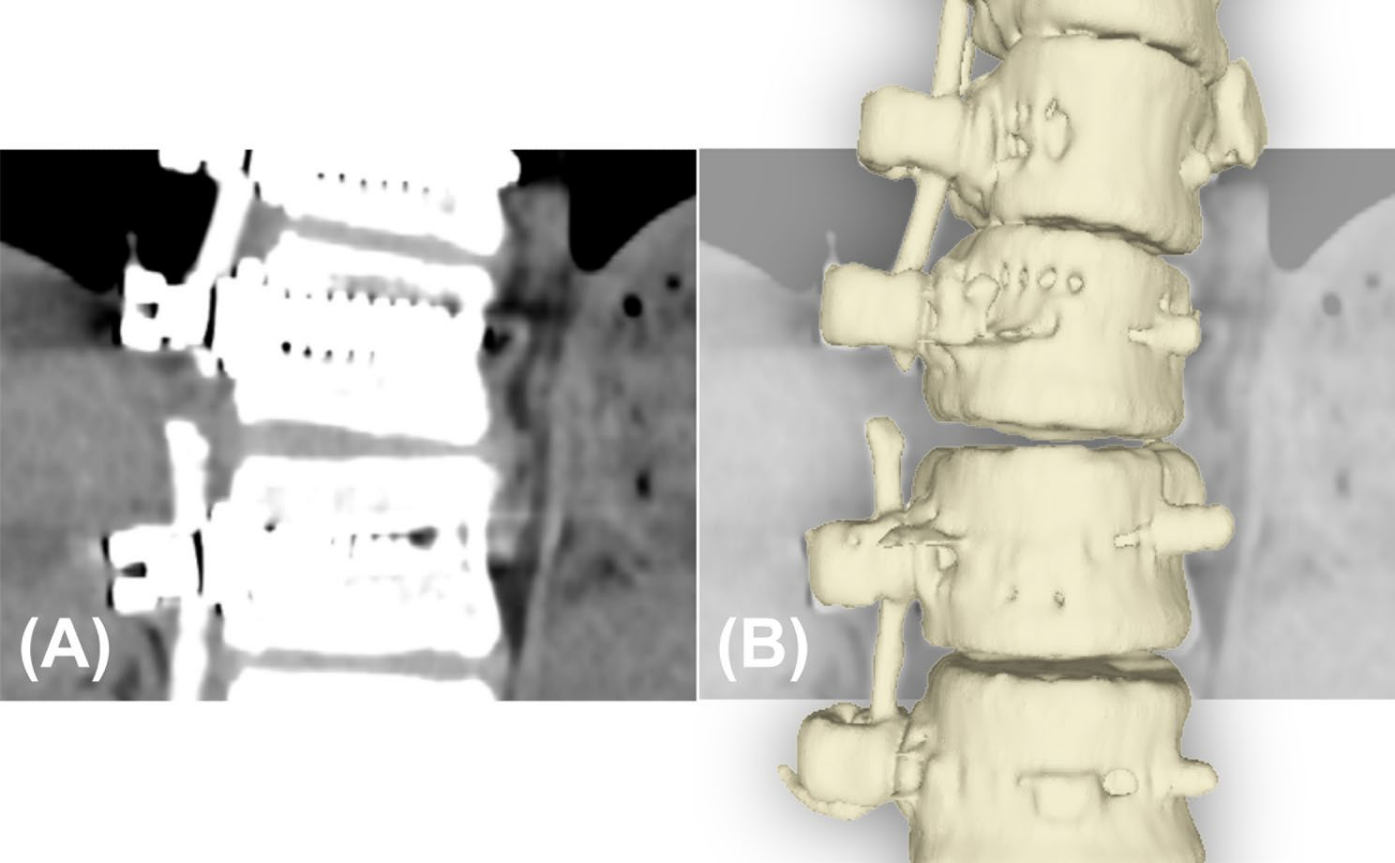
Example tether breakage located at T11–T12 screw–tether junction where the discontinuity between the tether segments on 2D CT slice is clearly visible (**A**), and its 3D reconstruction for visualization purposes (**B**)

Tether breakage on CT was defined as a clear discontinuity between tether segments on 2D CT scan slices (Fig. 3) [16].

### Study design and patients

This was a retrospective analysis of prospectively collected radiographic data of patients who underwent VBT at a single center between February 2019 and December 2020 for the treatment of Adolescent Idiopathic Scoliosis (AIS). Ethics approval was obtained from the local institutional review board (UW 19-002). All patients underwent surgery using implants from the same manufacturer (REFLECT™, Globus Medical, USA). Patients who have taken CT scans post-VBT were included in the study. Indications for scanning included the assessment of implant position and suspected tether breaks based on inter-screw index.

### Data and statistical analysis

Patient demographics (age at diagnosis, sex), skeletal maturity, curve type, and instrumented levels (UIV, LIV) were reviewed. Biplanar standing full-length radiographs (EOS imaging, Paris, France) taken at the first postoperative visit were compared with radiographs taken at the time of CT scanning (± 2 months). At each of the instrumented segments, Cobb angle of the instrumented curve, inter-screw distance, and angles were measured. To minimize bias, the measurements were conducted by two independent investigators (SW and MY) blinded to clinical and CT information. Inter-observer reliability was quantified using Intraclass Correlation Coefficient (ICC) with 95% confidence interval based on a two-way random-effects model [17]. Reliability levels of ICC were determined based on Koo et al. [18]. The diagnostic sensitivity of inter-screw index and inter-screw angle were compared based on their ability to identify tether breakages as confirmed on CT scans.

All CTs were assessed by two investigators (KC and SW) who were blinded to the radiographic findings and the tether breakage locations were identified. The distance between two broken ends of the tether at the diagnosed tether breakage locations was measured on 2D CT scan slices. To identify that the breakages observed in CT scans are not a by-product of the CT image quality and the other imaging artefacts, a known dimensional parameter, the diameter of the tether, was measured by two blinded investigators (SW, OG) at each broken level, and the standard error of measurement (SEM) was calculated by directly comparing it with the known diameter value of the tether [19]. Tether breakages with a higher breakage distance between the two broken ends than the standard error of measurement and the CT image resolution were accepted as the confirmed breakages.

All measurements were conducted on the local Picture Archiving and Communication System (PACS) using high image resolution and standardised calibration [20]. Statistical analysis was performed using IBM SPSS for Windows, Version. 27.0 (IBM Corp., Armonk, NY, USA).

## Results

### Demographic characteristics

Nine patients fit the eligibility criteria within the study period. 94 segments from 13 CT scans were reviewed. The mean age at surgery was 11.1 ± 1 years and the mean number of instrumented levels were 7.9 ± 1. There were 7 main thoracic curves, 2 double major curves, and 1 thoracolumbar curve. Mean duration between index surgery and CT scan was 16.3 months (range 2–36 months). Seven CT scans were performed for assessment of implant position, and six CT scans were performed because of suspected tether breakages.

### Tether breakage analysis

**Fig. 4:**
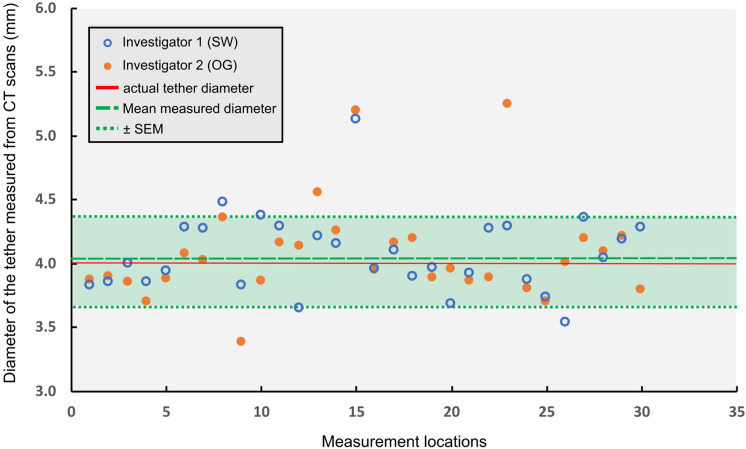
Tether diameter values at each broken level (cranial and caudal sides of the breakage) measured from the CT scans

On CT, tether breakages were observed in 15 segments in 7 of the patients that fit the eligibility criteria. SEM was calculated to be ± 0.35 mm (Fig. 4). The distance between two broken ends of the tether at the diagnosed tether breakage locations was 5.25 ± 2.1 mm (1.9–10.9 mm). All the tether breakages had a significantly bigger breakage distance between the two broken ends than the SEM and the CT image resolution. Therefore, all the breakages were accepted as confirmed breakages. No artefacts preventing the visualization of the tether were observed.

**Fig. 5:**
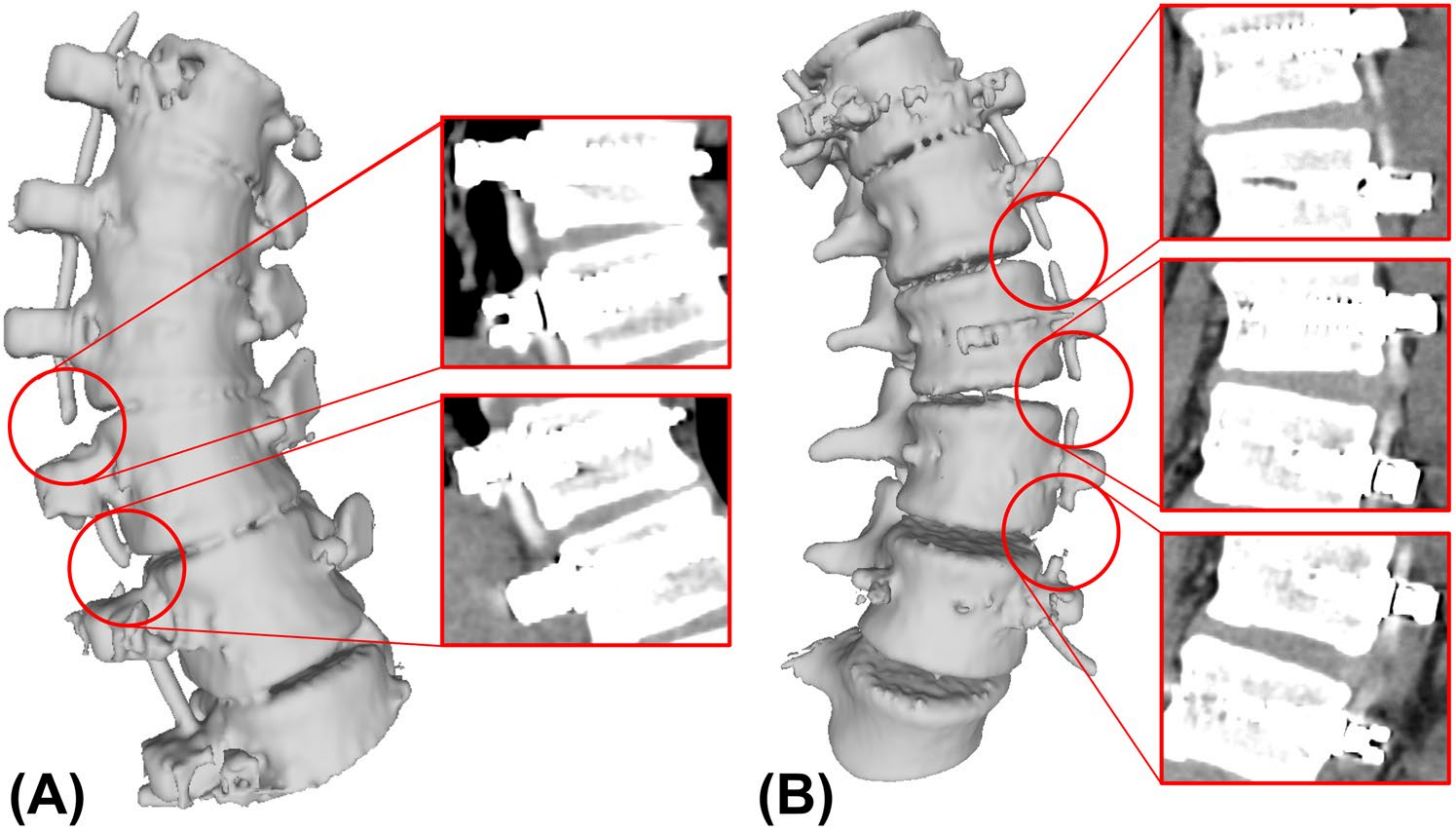
Tether breakage location examples observed on the thoracic spine (**A**) and lumbar spine (**B**), where the consecutive breakages at the screw–tether junction on thoracic spine and breakages at the mid-substance on lumbar spine are shown

Four of the breakages observed on CT were located at the apex of curves, while 11 of them were found 1–2 levels distal to the apex. No breakages were found above T9–T10. The rate of tether breakage was 15% for thoracic segments (9 out of 59) and 60% for lumbar segments (6 out of 10). Out of 15 tether breakages, 9 of them was located at the thoracic spine and failed at the screw–tether junction, while the remaining 6 were located at the lumbar spine and broke at the mid-substance (Fig. 5).

**Fig. 6:**
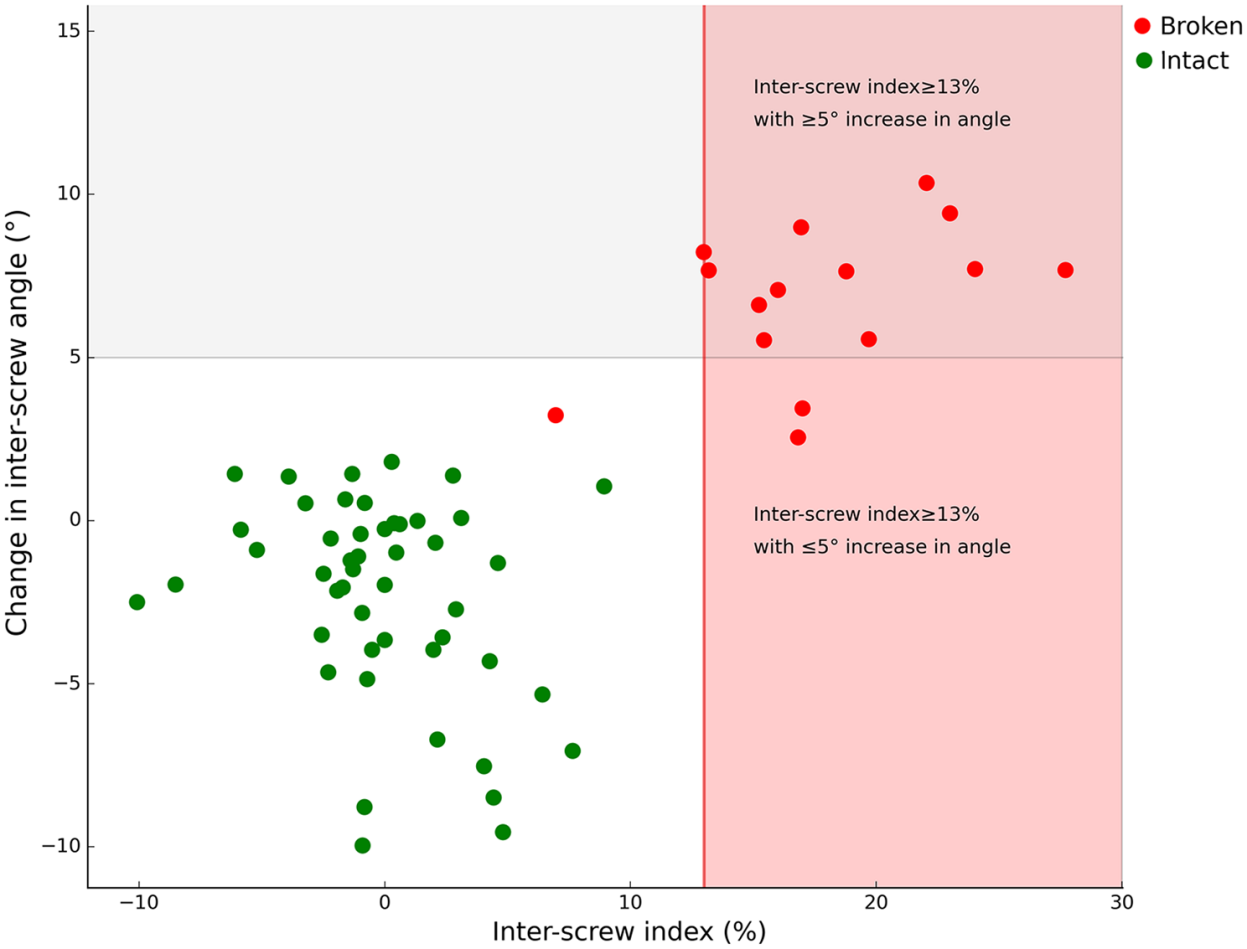
Scatter plot of inter-screw index and change in inter-screw angle of instrumented segments. Inter-screw index identified 14 broken tether segments, while inter-screw angle identified 12 broken segments out of 15

On plain radiographs, inter-screw index ≥ 13% was observed in 14 broken segments (sensitivity 93%), while ≥ 5° increase in inter-screw angle was observed in 12 broken segments (sensitivity 80%) (Fig. 6). No false positives were observed by either method. An overview of inter-screw measurements in patients with tether breakage is provided in Table 1.

**Table 1:** Overview of subjects with broken tethers

Study ID	Age at surgery	At time of confirmation of tether breakage					
		Risser/sanders	Cobb^a^	Time to breakage (months)	Confirmed broken levels	Inter-screw index ≥ 13% (Y/N)	Increase in inter-screw angle ≥ 5° (Y/N)
01	9	1/3	T6–L1: 12°	36	T9–T10	Y	Y
					T10–T11	Y	Y
02	10	1/3	T6–T12: 12°	31	T11–T12	Y	Y
03	11	3/4	T6–T12: 37°	30	T9–T10	Y	Y
			T12–L4: 46°		T10–T11	Y	N
					L1–L2	N	N
					L2–L3	Y	Y
					L3–L4	Y	Y
04	12	3/7	T6–T12: 25°	15	T9–T10	Y	Y
					T10–T11	Y	N
05	11	3/7	T10–L3: 45°	10	T12–L1	Y	Y
					L2–L3	Y	Y
06	12	2/7	T5–T11: 16°	14	T10–T11	Y	Y
07	11	(n/a)	T6–T11: 23°	18	T10–T11	Y	Y
			T11–L3: 19°		L2–L3	Y	Y

^a^Cobb angle of instrumented curve

**Fig. 7:**
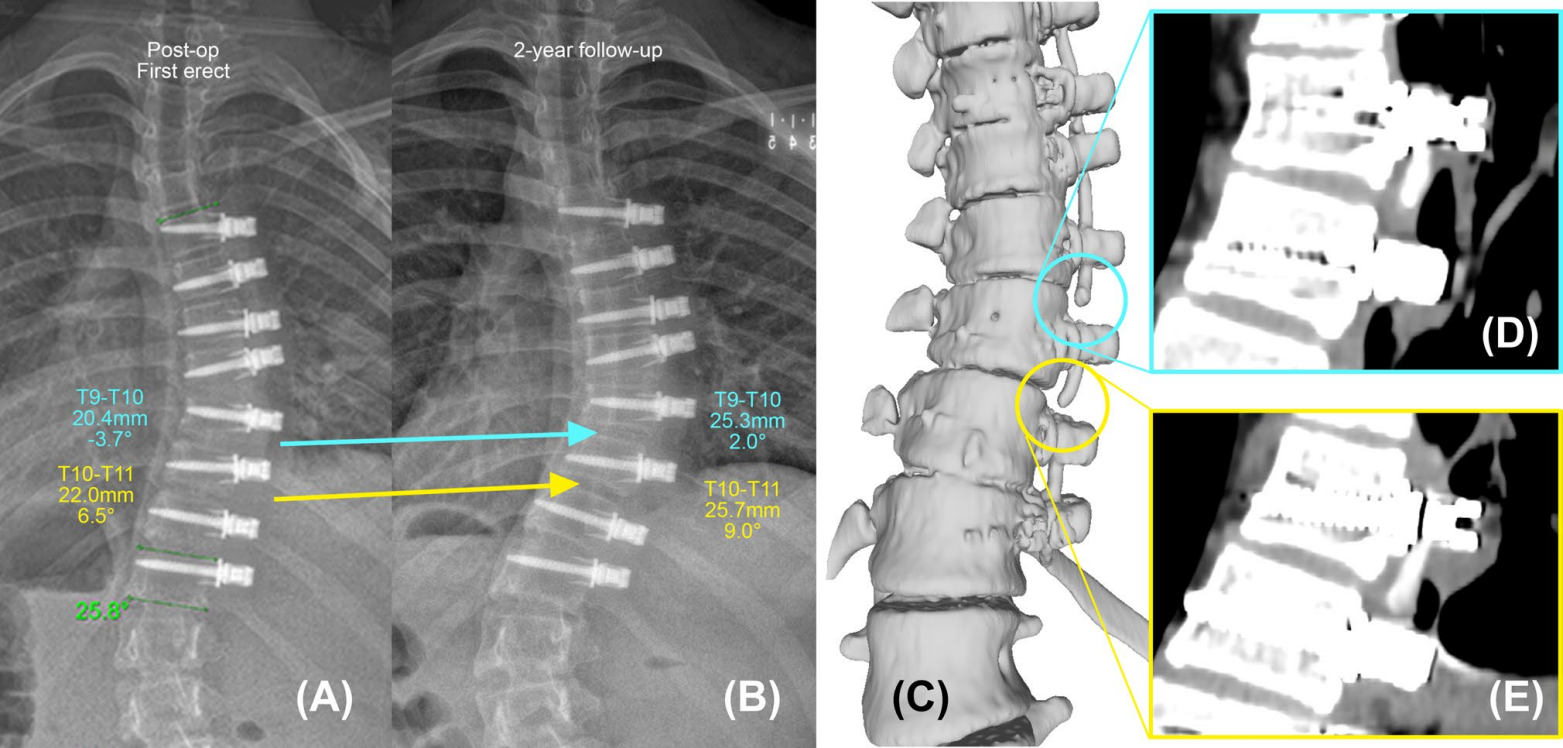
Example of a pair of consecutive breakages. The coronal radiographs taken post-op (**A**) and at the CT visit (**B**), the 3D reconstruction of the CT scan (**C**), and the 2D CT image slices where the upper (**D**) and lower (**E**) tether breakages located at T9–T10 and T10–T11 are shown. The upper breakage (blue) was identified by both radiographic criteria (inter-screw index = 24%, increase in inter-screw angle = 5.7), while the lower breakage (yellow) was only diagnosed by inter-screw index (inter-screw index = 17%, increase in inter-screw angle = 2.6)

There were 5 patients with breakages at consecutive levels. In identifying both breakages in consecutive breakages, inter-screw index showed 80% sensitivity (4 out of 5), while inter-screw angle showed 40% sensitivity (2 out of 5). In three pairs of consecutive breakages, inter-screw angle only identified one breakage from each pair. An example is shown in Figs. 5 and 7.

Inter-observer reliability ranged from 0.88 to 0.95 for change in inter-screw angle, and 0.89 to 0.96 for change in inter-screw distance (Table 2). Both methods showed good-to-excellent inter-observer reliability, with inter-screw index having a slightly higher ICC value.

**Table 2:** Inter-observer agreement of changes in inter-screw angle and inter-screw distance

	ICC	95% Cl
Change in inter-screw angle	0.924	[0.877; 0.953]
Change in inter-screw index	0.932	[0.942; 0.958]

## Discussion

Diagnosis of tether breakage through inter-screw angle assumes that angular change will always occur after the tether breaks. However, recent studies showed that angular change may not always be an indicator of the tether breakage. Trobish et al. reported the poor accuracy of this method and showed that tether breakage was not always accompanied by loss of correction [7]. In line with that, Baroncini et al. investigated the other factors that may be influencing the correction and reported that depending on timing tether breakage may significantly influence angular change [6]. In line with that, we clearly showed in this study that tethers structural integrity can be significantly influenced by extensive elongation without a corresponding change in angle, thus, making tether breakage diagnosis based on angular change less sensitive. Our results revealed that inter-screw angle is poor at diagnosing tether breakages where there is no loss of segmental correction, while inter-screw index successfully captured them with a higher accuracy.

Consecutive level tether breakages were reported previously on 7 out of 10 patients (70%) by Trobish et al. [13], and inter-screw angle was particularly poor in diagnosing these breakages. Similarly, we observed tether breakages at consecutive levels in our cohort, and the accuracy of inter-screw angle in identifying these breakages was only 40%. In three pairs of consecutive breakages, inter-screw angle only identified one breakage from each pair. We postulate that after one tether breaks, segmental loss of correction may occur in which the screw tilts away from the breakage, reducing the inter-screw angle of the adjacent segment that has yet to break. In addition, with one broken tether, there is loss of tension within the system that makes the next tether break less likely to tilt. Compared to inter-screw angle, inter-screw index successfully identified 80% of the breakages occurring at consecutive levels.

We observed a strong correlation between our clinical findings and our mechanical tests. Previously, we reported that the failure of the VBT construct occurs at the screw–tether junction and reported that this is the dominant failure mode [14]. Similarly, out of 15 confirmed tether breakages, 9 of them was located at the thoracic spine and consistently failed at the tether screw junction, implying that the mechanical principles observed in our tensile tests successfully explained the biomechanical reasons behind the tether breakages at the thoracic spine. On the other hand, the remaining six breakages located at the lumbar spine consistently occurring at the mid-substance revealed that the failure mode taking place at the lumbar spine is different than the thoracic spine. This distinction between the failure mechanisms of lumbar and thoracic VBT has been previously speculated to be due to increased material wear caused by the higher range of motion of the lumbar spine [7]. However, this has not been proven yet. Further study is suggested to investigate failure mechanisms taking place at the lumbar spine for further understanding of the biomechanics of VBT.

We observed that the tether breakages were diagnosed relatively early at 10, 14, and 15 months after the surgery, indicating that tether breakage could have occurred even earlier. According to Baroncini et al., tether breakages occurring within the first 12 months is significantly correlated with the loss of correction [6]. Moreover, Rushton et al. discussed that the factors that lead to early breakages may be different than those result in late breakages [21]. This highlights the importance of accurate and timely diagnosis, since factors that lead to breakage can only be investigated with certainty, if diagnosis can be made. To the author’s knowledge, this is one of the first studies highlighting early tether breakages.

A limitation of the current study is that only one VBT system is used. Previously, we reported that the failure at the screw–tether junction is a design-related complication [14]. Similarly, implant-related characteristics, such as the shape of the screw, strength of the tether, etc., has been reported as potential risk factors that could lead to breakage [13]. Therefore, further mechanical studies on other VBT systems from different manufacturers are needed to ascertain their behavior and the appropriateness of the 13% threshold value for the diagnosis of the tether breakage.

In summary, this is the first study to present inter-screw index as a rational indicator of tether breakage, and the first to offer an alternative diagnosis method of tether breakage independent of loss of correction. In our series of tether breakages confirmed on CT scans, a change in inter-screw distance, as represented by an increase in index of 13%, was able to identify more breakages than the inter-screw angle. Moreover, in the case of consecutive breakages, it was shown to be superior to the inter-screw angle. While larger studies would be needed to validate the generalizability of our results to other VBT systems, we propose that the concept of the inter-screw index has the potential to provide insights into the pattern and mechanism of tether breakage, as well as its effect on clinical outcome in the form of longitudinal studies.

## Data Availability

The datasets generated and/or analyzed during the current study are available from the corresponding author upon request.
